# A DISCUSSION ON THE STATE OF AFFAIRS ON DIRECT ACTION TO SUPPORT OLDER ADULTS DURING DISASTERS

**DOI:** 10.1093/geroni/igad104.0686

**Published:** 2023-12-21

**Authors:** Leah Haverhals, David Dosa

## Abstract

As the frequency and severity of disasters increases, supporting older adults before, during, and after disasters is paramount. Steps toward adequate disaster preparedness include ensuring older adults possess awareness of increased disaster and hazard events and understand their role in preparedness. This symposium will address the current state of affairs in the United States (US) on direct actions to support older adults before, during, and following major disasters. Additionally it will provide context on how continuing effects of climate change impact these efforts. Dr. Lisa Brown will share perspectives and describe efforts of the US National Advisory Committee on Seniors and Disasters. Dr. Sue Anne Bell will discuss the role of implementation science in improving the emergency preparedness in nursing homes. Dr. Lindsay Peterson will describe existing state policies around home and community-based service providers. Dr. Tamar Wyte-Lake will share directions the Department of Veterans Affairs is moving in to best support vulnerable populations, focusing on older Veterans. Dr. Karl Pillemer will address the role of older adults as active agents in climate change prevention and mediation. Dr. David Dosa will act as discussant. These presenters will provide important insights into ongoing efforts while highlighting areas in need of improvement and aligned action, with the hopes that this will generate important discussions well into the future. This is a Disasters and Older Adults Interest Group Sponsored Symposium.

